# Sensitivity to Tyrosine Kinase Inhibitors in a Human Philadelphia Chromosome-Positive (Ph+) Leukemia Model With the T315I-Inclusive Compound Mutation

**DOI:** 10.7759/cureus.76538

**Published:** 2024-12-28

**Authors:** Thao T Nguyen, Minori Tamai, Daisuke Harama, Keiko Kagami, Chiaki Komatsu, Shin Kasai, Koshi Akahane, Kumiko Goi, Takeshi Inukai

**Affiliations:** 1 Department of Pediatrics, University of Yamanashi, Chuo, JPN

**Keywords:** chronic myeloid leukemia (cml), compound mutation, crispr/cas9 system, human model, tyrosine kinase inhibitor (tki)

## Abstract

The T315I-inclusive compound mutation, the multiple mutations including the T315I mutation on the same BCR::ABL1 gene, confers resistance to diverse tyrosine kinase inhibitors (TKIs). Development of the F311I/T315I compound mutation has been reported in chronic myeloid leukemia patients who sequentially showed clinical resistance to imatinib and dasatinib. The establishment of a human leukemia model with the T315I-inclusive compound mutation remains an experimental challenge. Here, we introduced the F311I/T315I compound mutation into the intrinsic BCR::ABL1 gene of a human TKI-sensitive Philadelphia chromosome-positive leukemia cell line via homologous recombination using the CRISPR/Cas9 system and obtained three types of sublines: the F311I mutation alone, the T315I mutation alone, and the F311I/T315I compound mutation. The F311I subline was sensitive to dasatinib but moderately resistant to imatinib and nilotinib, while the T315I subline and the F311I/T315I subline were highly resistant to these TKIs. Notably, the T315I subline and the F311I/T315I subline were sensitive to therapeutic concentrations of ponatinib, although more resistant than the F311I subline. Moreover, the T315 subline and the F311I/T315 subline were sensitive to asciminib at therapeutic concentration, as was the F311I subline. This is the first human leukemia model in which the impact of the T315I-inclusive compound mutation on TKI sensitivity was directly confirmed.

## Introduction

Tyrosine kinase inhibitors (TKIs) target the BCR::ABL1 protein, which is derived from the Philadelphia chromosome (Ph). TKIs are the standard therapy for patients with chronic myeloid leukemia (CML) [1]. However, TKI resistance occurs in some patients due to point mutations of the BCR::ABL1 gene [2]. Imatinib and second-generation TKIs bind to the ATP-binding pocket of BCR::ABL1. Among residues constituting the ATP-binding pocket, threonine at codon 315 (T315) plays a critical role in stabilizing interactions between BCR::ABL1 and TKIs [2]. As a result, the T315I mutation induces severe resistance to imatinib and second-generation TKIs. Among the diverse BCR::ABL1 gene mutations, the compound mutation is defined as the presence of two (or more) mutations on the same allele, which is generally developed by sequential TKI treatment [3]. Notably, the T315I-inclusive compound mutations confer severe resistance to TKIs except ponatinib [3]. Among the T315I-inclusive compound mutations, the E255K/T315I compound mutation is clinically the most problematic. The intensive survey of the T315I-inclusive compound mutations in the literature identifies several key positions in the kinase domain of BCR::ABL1 beyond the T315 itself, and the most nearby key position of T315 in the DNA sequence is F311, which is substituted to F311I or F311L [3].

Development of the F311I/T315I compound mutation was reported in a CML patient who initially showed cytogenetic response to 400 mg daily imatinib [4]. The patient developed myeloid crisis along with the development of the F311I mutation, despite increasing the dose of imatinib to 600 mg. Notably, although the patient was tentatively controlled by 70 mg twice-daily dasatinib, the levels of the BCR::ABL1 transcripts gradually increased along with the acquisition of the T315I mutation, which was confirmed as the compound mutation of F311I/T315I. The clinical course of this case suggests that the F311I mutation alone confers resistance to imatinib but not dasatinib, while the F311I/T315I compound mutation further confers dasatinib resistance.

Ponatinib is a pan-BCR::ABL1 kinase inhibitor due to its potent activity against all single resistance mutations, including T315I mutant, as a result of effective hydrophobic contact with BCR::ABL1 having all of the single TKI resistant mutations [5]. In the clinical trials, the efficacy of ponatinib has been confirmed, including in patients with the T315I mutation [6]. Of clinical importance, ponatinib shows dose-dependent cardiovascular toxicity; hence, an elective dose reduction to 30 or 15 mg/dose is recommended [7,8]. When treated with a reduced 15 mg/dose of ponatinib, the serum concentration at steady state was reported to be approximately 25 nM, which could be insufficient to control the patients with the T315I mutation [9].

Anti-leukemic activities of TKIs have been generally tested in the murine Ba/F3 model [3], in which the native or mutated BCR::ABL1 cDNA is transfected. In the murine Ba/F3 model, the cells with BCR::ABL1 having the F311I mutation alone (F311I-BCR::ABL1) are significantly more resistant to imatinib and ponatinib, but almost equally sensitive to dasatinib, in comparison the cells with the native BCR::ABL1 (native-BCR::ABL1) [3]. Consistent with clinical findings, T315I-BCR::ABL1 cells and the cells with BCR::ABL1 having the compound mutation of F311I/T315I (F311I/T315I- BCR::ABL1) show severe resistance to imatinib and dasatinib [3]. Of note, F311I/T315I-BCR::ABL1 cells show more severe resistance to ponatinib in comparison with F311I-BCR::ABL1 cells and T315I-BCR::ABL1cells [3].

Asciminib is an allosteric inhibitor of BCR::ABL1, which binds to the myristoyl-binding site [10,11]. The myristoyl pocket of ABL1 is normally occupied by the myristoylated N-terminal of ABL1, which is an allosteric negative regulatory element of kinase activity. Notably, the myristoylated N-terminal of ABL1 is lost in BCR::ABL1 due to fusion. Asciminib mimics the myristate by binding to the myristoyl site and, subsequently, inhibits the kinase activity of BCR::ABL1 by inducing an inactive closed conformation. Accordingly, asciminib is active against BCR::ABL1, even with the T315I mutation (when treated with relatively higher concentrations of asciminib). In the murine Ba/F3 model [12], F311I-BCR::ABL1 cells and T315I-BCR::ABL1 cells are sensitive to asciminib, although less sensitive than the native-BCR::ABL1 cells. However, although the F311I/T315I compound mutation was not directly tested, representative BCR::ABL1 T315I-inclusive compound mutants uniformly conferred resistance to asciminib [12].

Despite its utility, the murine Ba/F3 cell system has several limitations. In the Ba/F3 model, the viral promoter instead of the intrinsic BCR gene promoter regulates the expression of the BCR::ABL1 gene. In the viral expression vector, the BCR::ABL1 cDNA lacks the 3'-untranslated region (UTR) and introns, which play a role in transcriptional and post-transcriptional regulation of gene expression. In this context, the interaction of microRNA through 3' UTR is reportedly involved in the post-transcriptional regulation of the BCR::ABL1 gene [13]. Moreover, alternative splicing of the BCR::ABL1 gene is reportedly involved in TKI resistance [14]. Furthermore, the reciprocal ABL1::BCR fusion gene is reportedly involved in leukemogenesis [15]. Under these circumstances, direct induction of the mutations into the intrinsic BCR::ABL1 gene in human leukemia cells could be another useful model system.

In the present study, to directly test the impact of F311I/T315I-BCR::ABL1 on TKI sensitivities in a human Ph+ leukemia model, we tried to introduce either the solo mutation of F311I or T315I, or the compound mutation of F311I/T315I, into the intrinsic BCR::ABL1 gene of the human Ph+ myeloid leukemia cell line by applying the homologous recombination (HR) of three corresponding templates using the CRISPR/Cas9 system. We successfully established the first human Ph+ leukemia model of the T315I-inclusive compound mutation and tested its TKI sensitivities.

## Materials and methods

Cell line

TCCS [16], which was previously provided by Dr Norio Komatsu, Juntendo University, Japan, was a human Ph+ leukemia cell line established from a CML myeloid blast crisis patient. The cells were maintained in RPMI1640 medium supplemented with 10% fetal calf serum (FCS).

Genome editing

The detailed protocol has been reported previously [17,18]. In brief, using the CRISPR design tool (CRISPR DESIGN, http://crispr.mit.edu), we designed sgRNA (5’-aactcagtgatgatatagaacgg-3’). Three template single-stranded oligodeoxynucleotides (ssODNs) were synthesized by Integrated DNA Technologies (Coralville, IA, USA). Sequeces of F311I-ssODN, T315I-ssODN, and F311I/T315I-ssODN were 5’-tgttgaagtcctcgttgtcttGTTGGCAGGGGTCTGCACCCgggagcccccgAtctaCatcatcaTtgagttcatgacctacgggaacctcctggactac-3’, 5’- tgttgaagtcctcgttgtcttGTTGGCAGGGGTCTGCACCCgggagcccccgttTtaCatcatcaTtgagttcatgacctacgggaacctcctggactac-3’, and 5’- tgttgaagtcctcgttgtcttGTTGGCAGGGGTCTGCACCCgggagcccccgAtctaCatcatcactgagttcatgacctacgggaacctcctggactac-3’, respectively. Before electroporation, cells were pre-treated with 10 nM of SCR7 (Cayman Chemical, Ann Arbor, MI, USA) for 24 hours. Subsequently, sgRNA and recombinant Cas9 (Integrated DNA Technologies) nuclease as a ribonucleoprotein complex with each template ssODN were transfected into 5 × 10^5^ of the pre-treated cells by electroporation using the Neon electroporation transfection system (ThermoFisher Scientific, Waltham, MA, USA). The electroporated cells were transferred to a 96-well plate and cultured in the presence of 10 nM of SCR7 for 48 hours, and the cells were further expanded in the absence of imatinib for seven days. The cells were then cultured in the presence of 1 μM of imatinib for 14 days. The obtained imatinib-resistant sublines were transferred to culture flasks and expanded in the absence of imatinib for further experiments.

Direct sequencing and thymine-adenine (TA) cloning

Genomic DNA was extracted using PureLink Genomic DNA Mini Kit (ThermoFisher Scientific) and the polymerase chain reaction (PCR) of the BCR::ABL1 gene was performed using the forward (5’-ccacacgagcacagtctcag-3’) and reverse (5’-aactcagtgatgatatagaacgg-3’) primers. Direct sequencing of each PCR product was performed using the forward primer. For TA cloning, genomic PCR products were cloned using the TOPO^TM^ TA Cloning^TM^ Kit (ThermoFisher Scientific). Plasmid DNA was extracted from the colonies using a QIA Plasmid Mini Kit (Qiagen, Hilden, Germany), and high-quality samples were subjected to direct sequencing.

Drug sensitivity assay

To determine the sensitivities to TKIs and asciminib of three sublines and their parental cells, an alamarBlue cell viability assay (Bio-Rad Laboratories, Hercules, CA) was performed. A total of 1-5 × 10^5^ cells were plated into a 96-well flat-bottom plate in triplicate and cultured for 72 hours [12] in the presence or absence of each drug. Imatinib (S2475) and asciminib (S8555) were purchased from Selleck Chemicals (Tokyo, Japan), and ponatinib (CS-0204) was purchased from Chem Scene (Monmouth Junction, NJ). Nilotinib and dasatinib were kindly provided by Dr Tetsuzo Tauchi, Tokyo Medical University, Japan. After a six-hour additional incubation with alamarBlue, absorbance at 570 nm was monitored by a microplate spectrophotometer using 600 nm as a reference wavelength. Cell survival was calculated by expressing the ratio of the optical density of the treated wells to that of the untreated wells as a percentage. The concentration of the drug required to reduce the viability of treated cells to 50% of untreated cells was calculated, and the median of three independent assays was determined as the 50% inhibitory concentration (IC50) for each subline.

Statistical analysis

Statistical analyses were undertaken using Jamovi software version 2.3.16 (Jamovi, Sydney, Australia).

## Results

Introduction of the T315I-inclusive compound mutation into the human Ph+ leukemia cell line

**Figure 1:**
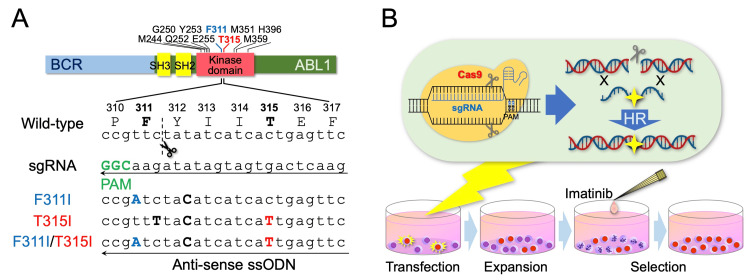
Introduction of the T315I-inclusive compound mutation into a human Ph+ leukemia cell line. A: Schematic diagram of the sgRNA and the ssODN. In the top panel, 10 key positions of the compound mutation of the BCR::ABL1 protein, including F311 and T315, are indicated. In the bottom panel, the top indicates wild-type amino acid and nucleotide sequences, the middle indicates the sequence of sgRNA, and the bottom indicates the sequence of three ssODN templates. In the sgRNA sequence, the protospacer adjacent motif (PAM) site is indicated in bold green. The scissors icon and dotted line indicate the cleave site of the Cas9 nuclease. Mutated nucleotides are indicated in bold capitals. B: Transfection and selection workflow.

We tried to establish three mutated sublines having the F311I or T315I alone or the F311I/T315I compound mutation by HR using the CRISPR/Cas9 system (Figure 1A). We targeted ‘CCG’ at P310 as the protospacer adjacent motif (PAM) site in the anti-sense direction of sgRNA. We designed three templates of ssODN. For the F311I-BCR::ABL1, template ssODN has a substitution from ‘TTC’ at F311 to ‘ATC’. For the T315I-BCR::ABL1, template ssODN has the substitution from ‘ACT’ at T315 to ‘ATT’. For the F311I/T315I-BCR::ABL1, template ssODN has substitutions from ‘TTC’ at F311 to ‘ATC’ and ‘ACT’ at T315 to ‘ATT’. Moreover, in all three ssODN templates, we designed an additional silent mutation at Y312 from ‘TAT’ to ‘TAC’ to reduce the opportunities for re-cutting of the mutated target site by Cas9. We transfected each ssODN template and the sgRNA with recombinant Cas9 protein by electroporation into the TCCS cells that were pre-treated with SCR7 (an inhibitor for non-homologous end-joining) to enhance HR efficiency [19]. TCCS is a human Ph+ myeloid cell line established from the myeloid crisis of CML [16]. We previously confirmed that TCCS has four alleles of the BCR::ABL1 fusion gene without an intact allele of the ABL1 gene by using dual-color fluorescence in situ hybridization analysis [18]. After electroporation, the cells were cultured in the absence of imatinib for seven days for expansion of the cells (Figure 1B). Subsequently, the cells were cultured for 14 days for selection in the presence of imatinib at 1 μM, which effectively killed the parental cells of TCCS (Figure 1B). We obtained the imatinib-resistant subline for each of the three transfections.

Establishment of subline with the T315I-inclusive compound mutation

**Figure 2:**
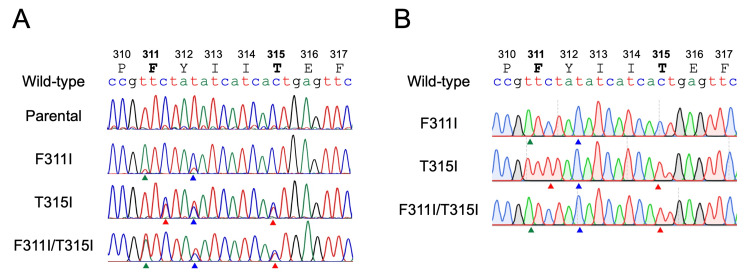
Establishment of the subline acquired the T315I-inclusive compound mutation. A: Genomic sequencing of the polymerase chain reaction (PCR) products of three imatinib-resistant sublines transfected with the F311I, T315I, and F311I/T315I templates as well as parental cells. Wild-type amino acids and genomic sequences are indicated at the top. Arrowheads indicate mutated nucleotides. B: Sanger sequencing of the PCR products after thymine-adenine (TA) cloning in three imatinib-resistant sublines. Sequences of each mutated allele are indicated. Arrowheads indicate mutated nucleotides.

From each imatinib-resistant subline, we extracted genomic DNA and performed direct Sanger sequencing of the PCR products of the BCR::ABL1 fusion gene (Figure 2A). In the imatinib-resistant subline transfected with the F311I-BCR::ABL1 template, higher peaks of two mutated nucleotides, which corresponded to the F311I-BCR::ABL1 and the silent mutation of Y312, were observed with lower peaks of the wild-type nucleotides. Next, in the imatinib-resistant subline transfected with the T315I-BCR::ABL1 template, the peaks of two mutated nucleotides, which corresponded to the silent mutation of Y312 and the T315I-BCR::ABL1, were as high as the peaks of the wild-type nucleotides. Similarly, in the imatinib-resistant subline transfected with the F311I/T315I-BCR::ABL1 template, peaks of three mutated nucleotides, which corresponded to the F311I-BCR::ABL1, the silent mutation of Y312, and the T315I-BCR::ABL1, were as high as the peaks of the wild-type nucleotides. We then performed Sanger sequence analysis after the TA-cloning of the PCR products of three sublines (Figure 2B). We confirmed that each mutation was introduced in the same allele in all three sublines. Of importance, in the sublines transfected with the F311I/T315I-BCR::ABL1 template, both F311I and T315I were introduced into the same allele, indicating the acquisition of compound mutation. Moreover, the silent mutation of Y312 was always acquired in the same allele, indicating that these three types of mutations were integrated into the same BCR::ABL1 allele as a result of HR with the corresponding template ssODN. Since TCCS has four alleles of the BCR::ABL1 fusion gene, these observations indicated that the imatinib-resistant sublines of the F311I-BCR::ABL1, T315I-BCR::ABL1, and F311I/T315I-BCR::ABL1 acquired the intended mutations in three, two, and two out of four alleles of the BCR::ABL1 fusion gene, respectively.

TKI sensitivity of the subline acquiring the T315I-inclusive compound mutation

**Figure 3:**
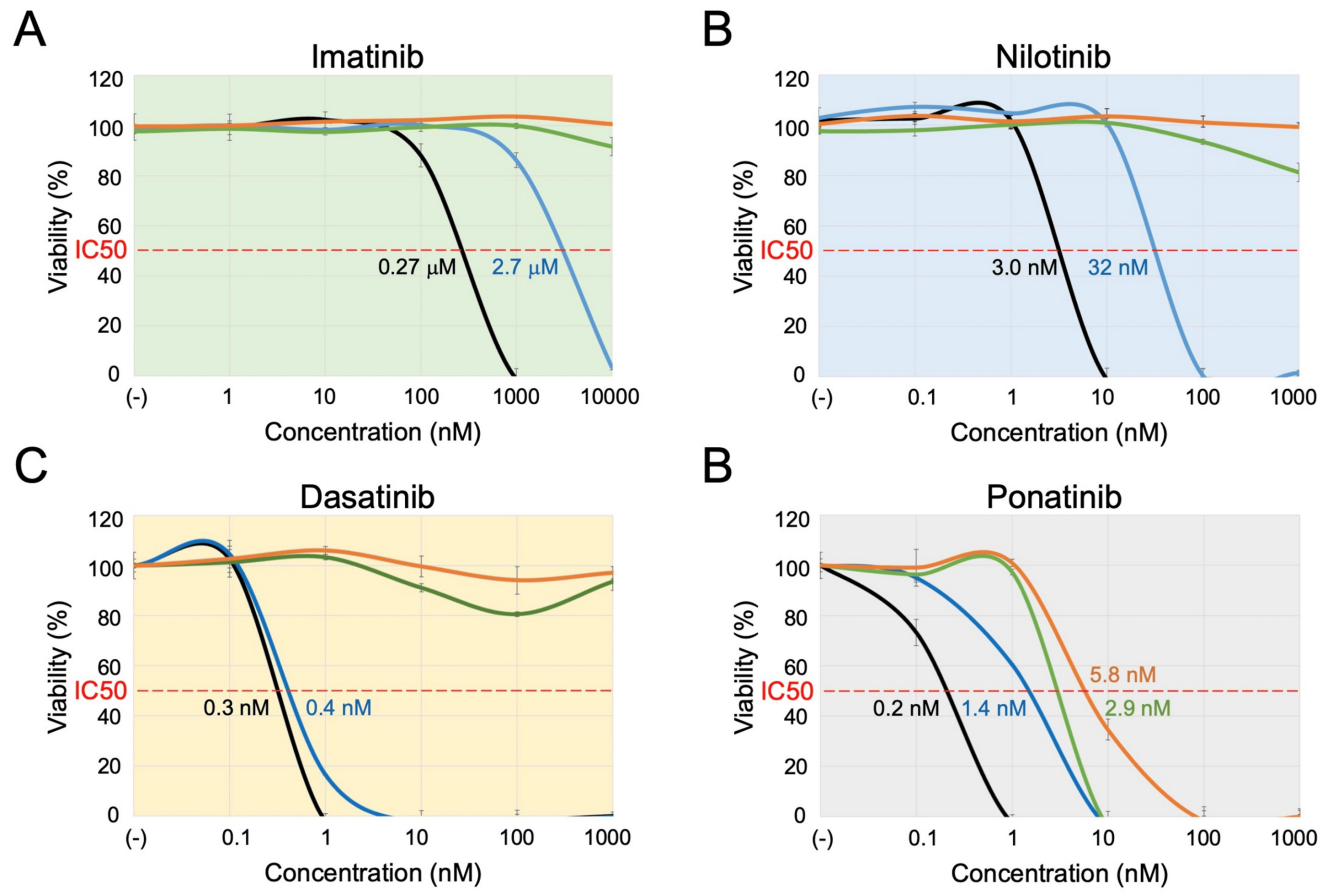
Dose-response curves of TKIs. A: Dose-response curve of imatinib. B: Dose-response curve of nilotinib. C: Dose-response curve of dasatinib. D: Dose-response curve of ponatinib. The black, blue, green, and orange lines indicate dose-response curves in the parental cells, F311I-BCR::ABL1 subline, T315I-BCR::ABL1 subline, and F311I/T315I-BCR::ABL1 subline, respectively. The horizontal and vertical axes indicate the concentrations of TKIs and viability, respectively. Error bars indicate standard errors in the triplicated analysis. The IC50 value of each subline is indicated when sensitive. TKIs: tyrosine kinase inhibitors; IC50: half maximal inhibitory concentration

We evaluated the sensitivities to four TKIs in three sublines in comparison with the parental cells using the alamarBlue assay (Table 1 and Figure 3). Cells were cultured in the presence of imatinib up to 10 μM (therapeutic concentrations: 2.4-5.2 μM) [20], nilotinib up to 1 μM (1-2.3 μM) [21], dasatinib up to 1 μM (0.012-0.49 μM) [22], and ponatinib up to 1 μM (0.023-0.14 μM) [5,9] for 72 hours. Regarding imatinib sensitivity (Table 1 and Figure 3A), the parental cells were sensitive (IC50: 0.27 μM). Although significantly more resistant than the parental cells, the F311I-BCR::ABL1 subline was moderately sensitive to imatinib (2.7 μM). In contrast to the F311I-BCR::ABL1 subline, both the T315I-BCR::ABL1 subline and the F311I/T315I-BCR::ABL1 subline were highly resistant to imatinib up to 10 μM. Regarding nilotinib sensitivity (Table 1 and Figure 3B), the parental cells were highly sensitive (IC50: 3.0 nM). Although significantly more resistant than the parental cells, the F311I-BCR::ABL1 subline was moderately sensitive to nilotinib (32 nM). In contrast to the F311I-BCR::ABL1 subline, both the T315I-BCR::ABL1 subline and the F311I/T315I-BCR::ABL1 subline were highly resistant to nilotinib up to 1 μM. Regarding dasatinib sensitivity (Table 1 and Figure 3C), the parental cells (IC50: 0.3 nM) and the F311I-BCR::ABL1 subline (0.4 nM) were almost equally sensitive. In contrast, both the T315I-BCR::ABL1 subline and the F311I/T315I-BCR::ABL1 subline were highly resistant to dasatinib up to 1 μM. Finally, regarding ponatinib sensitivity (Table 1 and Figure 3D), the parental cells were highly sensitive (IC50: 0.2 nM). Although more resistant than the parental cells, the F311I-BCR::ABL1 subline was sensitive to ponatinib (1.4 nM). Moreover, although more resistant to the F311I-BCR::ABL1 subline, the T315I-BCR::ABL1 subline was sensitive to ponatinib (2.9 nM). Of note, although more resistant than the T315I-BCR::ABL1 subline, the F311I/T315I-BCR::ABL1 subline was sensitive to ponatinib (5.8 nM).

**Table 1:** Summary of TKI and asciminib sensitivities. Sensitivities in the reported clinical case [4], in a murine Ba/F3 model [3,12], and in a human Ph+ myeloid leukemia model (TCCS cell line) of the present study are summarized. TKI: tyrosine kinase inhibitor; IC50: half maximal inhibitory concentration; NA: not applied; NT: not tested

		Imatinib	Nilotinib	Dasatinib	Ponatinib	Asciminib	Reference
	BCR::ABL1	C_max_: 2.4-5.2 μM	C_max_: 1-2.3 μM	C_max_: 12-490 nM	C_max_: 23-140 nM	C_max_: 1.7-3.9 μM	
Clinical case	Native	Sensitive (400 mg daily)	NA	NA	NA	NA	Blood 2008 [4]
	F311I	Resistant (600 mg daily)	NA	Sensitive (70 mg twice daily)	NA	NA	
	F311I/T315I	NA	NA	Resistant (70 mg twice daily)	NA	NA	
Murine Ba/F3	Native	IC50: 0.8 μM	IC50: 70 nM	IC50: 3 nM	IC50: 4 nM	IC50: 3.8 nM	Cancer Cell 2014 [3] Cancer Cell 2019 [12]
	F311I	IC50: 6 μM	IC50: 200 nM	IC50: 3 nM	IC50: 12 nM	IC50: 142 nM	
	T315I	IC50: >10 μM	IC50: >10 μM	IC50: >800 nM	IC50: 30 nM	IC50: 29 nM	
	F311I/T315I	IC50: >10 μM	IC50: >10 μM	IC50: >800 nM	IC50: 200 nM	NT	
Human TCCS	Native	IC50: 0.27 μM	IC50: 3 nM	IC50: 0.3 nM	IC50: 0.2 nM	IC50: 1.8 nM	Present study
	F311I	IC50: 2.7 μM	IC50: 32 nM	IC50: 0.4 nM	IC50: 1.4 nM	IC50: 29 nM	
	T315I	IC50: >10 μM	IC50: >1 μM	IC50: >1 μM	IC50: 2.9 nM	IC50: 34 nM	
	F311I/T315I	IC50: >10 μM	IC50: >1 μM	IC50: >1 μM	IC50: 5.8 nM	IC50: 40 nM	

Asciminib sensitivity of subline acquired the T315I-inclusive compound mutation

**Figure 4:**
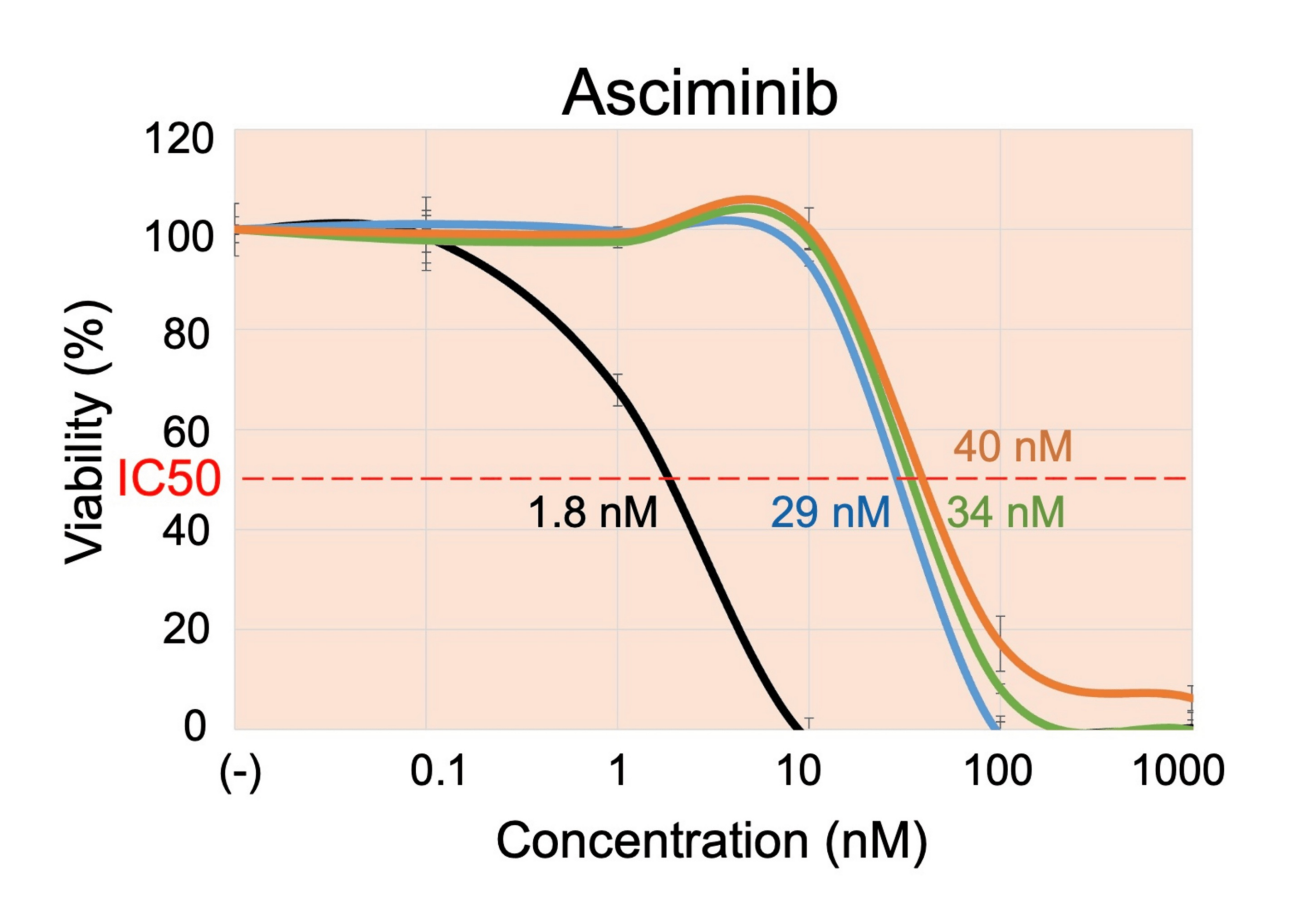
Dose-response curves of asciminib. The black, blue, green, and orange lines indicate dose-response curves in the parental cells, F311I-BCR::ABL1 subline, T315I-BCR::ABL1 subline, and F311I/T315I-BCR::ABL1 subline, respectively. The horizontal and vertical axes indicate the concentrations of asciminib and viability, respectively. Error bars indicate standard errors in the triplicated analysis. The IC50 value of each subline is indicated. IC50: half maximal inhibitory concentration

We also evaluated the asciminib sensitivity in the three sublines in comparison with the parental cells using the alamarBlue assay (Table 1 and Figure 4). Cells were cultured in the presence of asciminib up to 1 μM (therapeutic concentrations: 1.7-3.9 μM) [23]. The parental cells were highly sensitive to asciminib (IC50: 1.8 nM). Although more resistant than the parental cells, the F311I-BCR::ABL1 subline was sensitive to asciminib (28 nM). Of note, both the T315I-BCR::ABL1 subline (34 nM) and the F311I/T315I-BCR::ABL1 subline (40 nM) were almost equally sensitive to asciminib as the F311I-BCR::ABL1 subline.

## Discussion

In the present study, we successfully introduced F311I, T315I, and F311I/T315I mutations into the intrinsic BCR::ABL1 gene of TCCS (a TKI-sensitive human Ph+ myeloid leukemia cell line) using the CRISPR/Cas9 system. These mutations were introduced as a result of HR with the ssODN templates since the silent mutation of Y312 was introduced simultaneously. In human Ph+ leukemia cell lines, amplification of the BCR::ABL1 gene is generally observed [24], and TCCS has four alleles of the BCR::ABL1 gene. The F311I-BCR::ABL1 subline, the T315I-BCR::ABL1 subline, and the F311I/T315I-BCR::ABL1 subline acquired mutations in three, two, and two out of four alleles, respectively. Importantly, in the F311I/T315I-BCR::ABL1 subline, both the F311I and T315I mutations were introduced in the same alleles, indicating a compound mutation. Although the numbers of integrated alleles were smaller (two alleles vs. three alleles), the T315I-BCR::ABL1 subline and the F311I/T315I-BCR::ABL1 subline revealed higher levels of resistance to imatinib, nilotinib, and dasatinib compared with the F311I-BCR::ABL1 subline. Thus, as expected, our human TCCS model indicates that the T315I and the F311I/T315I mutations confer higher resistance to imatinib, nilotinib, and dasatinib than the F311I mutation.

This is the first human leukemia model in which the significance of the T315I-inclusive compound mutation in TKI sensitivity was confirmed. However, there are several limitations in this study. First, we applied HR to introduce the T315I-inclusive compound mutation. Thus, in the present study, we could not test the significance of the E255K/T315I mutation, which is clinically the most problematic T315I-inclusive compound mutation, because of a relatively large distance in the DNA sequence between E255 and T315. Second, we evaluated the TKI sensitivities in the standard drug sensitivity assay, in which cells are incubated with TKI for 72 hours. Thus, since exposure to TKI was not performed in the pharmacological condition, drug sensitivities in the present study might not properly reflect the sensitivities in the clinical setting.

In our human model, we confirmed that the F311I, T315I, and F311I/T315I mutations conferred resistance to ponatinib. Notably, although more resistant than the parental cells that have the native-BCR::ABL1 (IC50: 0.2 nM), the F311I-BCR::ABL1 subline (1.4 nM), T315I-BCR::ABL1 subline (2.9 nM), and F311I/T315I-BCR::ABL1 subline (5.8 nM) were sensitive to ponatinib (Table 1). In the clinical setting, elective dose reduction of ponatinib to 30 or 15 mg/dose is recommended due to its cardiovascular toxicity [7,8]. When treated with a reduced dose of ponatinib at 15 mg, the serum concentration at steady state was reported to be approximately 25 nM [9]. Thus, the IC50 values in all three sublines, including the F311I/T315I-BCR::ABL1 subline were lower than the serum concentration of ponatinib in the reduced doses. In contrast to our observations, the murine Ba/F3 model suggests that the reduced doses of ponatinib might be ineffective against T315I-BCR::ABL1 (IC50: 30 nM) and F311I/T315I-BCR::ABL1 (200 nM) (Table 1) [3].

Regarding the differences in ponatinib sensitivities between the murine Ba/F3 model [3] and our human TCCS model (Table 1), the native or the mutated cDNA of the BCR::ABL1 fusion gene is retrovirally transduced in the murine Ba/F3 model. Thus, Ba/F3 cells with the F311I-BCR::ABL1, the T315I-BCR::ABL1, and the F311I/T315I-BCR::ABL1 express mutated BCR::ABL1 alone without native-BCR::ABL1. In contrast, our human TCCS model has four alleles of the BCR::ABL1 fusion gene, and the F311I-BCR::ABL1 subline, the T315I-BCR::ABL1 subline, and the F311I/T315I-BCR::ABL1 subline acquired mutations in three, two, and two alleles, respectively. Thus, all three mutated sublines of TCCS also expressed native BCR::ABL1 protein to some degree as well as the mutated protein. Since BCR::ABL1 reportedly acts as a homotetramer-two dimers stack onto each other at the oligomerization domain of BCR [25,26], most BCR::ABL1 tetramers in our mutated sublines could be composed of both native and mutated BCR::ABL1. In this situation, our TCCS model might somehow underestimate the impact of the F311I/T315I mutation on TKI sensitivity compared with the actual Ph+ leukemia cells that have the mutation in only one BCR::ABL1 allele.

In the Ba/F3 model [12], the asciminib sensitivity of the F311I/T315I cells was not directly tested in the previous report. In our TCCS models (Table 1), although more resistant than the parental cells, all three mutated sublines of TCCS (including the F311I/T315I-BCR::ABL1 subline) were almost equally sensitive to asciminib at nanomolar ranges (IC50: 29-40 nM). Since the C_max_ of asciminib at the steady-state was reportedly 1.7-3.9 μM [23], our observations suggest that standard doses of asciminib in the clinical setting are effective for all three mutated sublines, including the F311I/T315I-BCR::ABL1 subline. Since BCR::ABL1 tetramers in our TCCS model are supposedly composed of native and mutated BCR::ABL1 proteins, our results suggest that asciminib might still be active in such chimeric tetramers. In the clinical course of CML, the double Ph chromosome has been reported in disease progression [27,28], and the acquisition of the T315I mutation in these cases reportedly leads to further resistance to imatinib [29,30]. Our observations suggest that asciminib might be effective when these cases with multiple copies of the BCR::ABL1 gene acquire the point mutations including the T315I-inclusive compound mutations in one of multiple alleles.

## Conclusions

We established a human Ph+ leukemia model of the F311I/T315I compound mutation as well as F311I alone and T315I alone via HR of the ssODN template using the CRISPR/Cas9 system. This is the first application of the CRISPR/Cas9 system to establish a human Ph-positive leukemia model of the T315I-inclusive compound mutation. Using this model, we evaluated its significance on the TKI sensitivity and confirmed induction of more severe resistance to TKIs including ponatinib, but not asciminib, by F311I/T315I compound mutation in comparison with F311I alone and T315I alone. Our strategy of gene editing could be useful for the establishment of human leukemia models for other types of T315I-inclusive compound mutations.
